# Melanoblasts Populate the Mouse Choroid Earlier in Development Than Previously Described

**DOI:** 10.1167/iovs.61.10.33

**Published:** 2020-08-14

**Authors:** Paul G. McMenamin, Graham T. Shields, Yashar Seyed-Razavi, Helen Kalirai, Robert H. Insall, Laura M. Machesky, Sarah E. Coupland

**Affiliations:** 1 Department of Anatomy and Developmental Biology, School of Biomedical Sciences, Faculty of Medicine, Nursing and Health Sciences, Monash University, Clayton, Victoria, Australia; 2 Liverpool Ocular Oncology Research Group, Department of Molecular and Clinical Cancer Medicine, Institute of Translational Medicine, University of Liverpool, Liverpool, United Kingdom; 3 Liverpool Clinical Laboratories, Liverpool University Hospitals NHS Foundation Trust, Liverpool, United Kingdom; 4 CRUK Beatson Institute, Bearsden, University of Glasgow, Glasgow, G61 1BD, United Kingdom; 5 Institute of Cancer Sciences, University of Glasgow, Glasgow, United Kingdom

**Keywords:** melanoblasts, melanocytes, uveal tract, choroid, development, migration

## Abstract

**Purpose:**

Human choroidal melanocytes become evident in the last trimester of development, but very little is known about them. To better understand normal and diseased choroidal melanocyte biology we examined their precursors, melanoblasts (MB), in mouse eyes during development, particularly their relation to the developing vasculature and immune cells.

**Methods:**

Naïve B6(Cg)-Tyr^c-2J^/J albino mice were used between embryonic (E) day 15.5 and postnatal (P) day 8, with adult controls. Whole eyes, posterior segments, or dissected choroidal wholemounts were stained with antibodies against tyrosinase-related protein 2, ionized calcium binding adaptor molecule-1 or isolectin B4, and examined by confocal microscopy. Immunoreactive cell numbers in the choroid were quantified with Imaris. One-way ANOVA with Tukey's post hoc test assessed statistical significance.

**Results:**

Small numbers of MB were present in the presumptive choroid at E15.5 and E18.5. The density significantly increased between E18.5 (381.4 ± 45.8 cells/mm^2^) and P0 (695.2 ± 87.1 cells/mm^2^; *P* = 0.032). In postnatal eyes MB increased in density and formed multiple layers beneath the choriocapillaris. MB in the periocular mesenchyme preceded the appearance of vascular structures at E15.5. Myeloid cells (Ionized calcium binding adaptor molecule-1-positive) were also present at high densities from this time, and attained adult-equivalent densities by P8 (556.4 ± 73.6 cells/mm^2^).

**Conclusions:**

We demonstrate that choroidal MB and myeloid cells are both present at very early stages of mouse eye development (E15.5). Although MB and vascularization seemed to be unlinked early in choroidal development, they were closely associated at later stages. MB did not migrate into the choroid in waves, nor did they have a consistent relationship with nerves.

Melanoblasts (MB) are the embryonic precursors of pigment-producing melanocytes present in the eyes, ears, meninges, heart, and skin.^1^ They are thought to arise directly from neural crest cells (NCC) and/or secondarily from other NCC-derived cells, Schwann cell precursors (SCP). Subsequently, MB colonize different body sites as a result of differing signaling molecules.^2^ Although the timing and molecular mechanisms involved in migration of skin MB are well-established, there is a huge gap in our understanding of noncutaneous MB migration, for example, into the eyes, where they are present in the uveal tract (i.e., choroid, ciliary body, and iris). In this study, we concentrate on choroidal MB biology and migration, with the overall objective that a better understanding of these developmental processes will augment our comprehension of neoplastic melanocytes in the uvea and their dissemination during metastasis.

The mature mammalian choroid comprises blood vessels, melanocytes, fibroblasts, resident immunocompetent cells, and supporting collagenous and elastic connective tissue.^3^^,^^4^ Its highly vascularized nature reflects its main function, that is, the supply of oxygen and nutrients to either the outer retina in species that possess a retinal vasculature or to the entire retina in species that lack a retinal blood supply. Other functions include thermoregulation, intraocular pressure modulation, and aqueous humor drainage via the uveoscleral pathway.^3^^,^^5^ The numerous melanocytes present in the human choroidal stroma are distributed below the choriocapillaris in the vascular layers of Haller and Sattler, some being perivascular in location.^3^ They are also present in the lamina fusca of the suprachoroid, where they take on a fusiform morphology. Apart from providing pigmentation to absorb light, it is unclear what other functions choroidal melanocytes may perform.

Early histologic and ultrastructural observations of developing choroid in primates led to the dogma that MB do not migrate into the choroid until the third trimester.^6^^–^^8^ These studies observed the appearance of melanin in the RPE as early as 60 days in the rhesus monkeys (total gestation period 166 days) and week 7 in humans, but “stromal pigment cells” (i.e., melanocytes) did not appear in the choroid until late in gestation—154 days in rhesus monkeys^7^ and week 27 in humans.^8^ The authors of these studies did not mention whether premelanosomes, stages I to II melanosomes that do not yet contain melanin,^9^ could be seen at earlier time points.

Proteomic analysis of melanosomes shows that of the approximately 1500 proteins appearing in all stages of melanosome maturation, twelve are specifically localized in melanosomes, including tyrosinase (TYR), tyrosinase-related protein 1 (Tyrp1/TRP1), and tyrosinase-related protein 2 (Tyrp2/TRP2).^10^ In a study examining mammalian MB differentiation and migration in the eye, ear and Harderian Gland, TRP2 (also known as Dopachrome Tautomerase), was expressed as early as E10 in migrating mouse MB, 4 days earlier than the other markers, TYR and TRP1.^11^ Expression of TRP2 was initially extensively studied by in situ hybridization to map the migration of MB from the neural crest in mouse tissue towards the eyes and skin.^11^ The development of an anti-TRP2 antibody^12^ has proven valuable in extending such studies, but to date has not been applied to investigate the MB migration into the developing eye.

As part of a wider investigation into the biology of choroidal MB/melanocytes, with relevance to better understanding uveal melanoma (UM) development and spread (and thus patient outcomes), the present study was designed to define the appearance of MB in the developing mouse eye, particularly in relation to the vasculature, nerves and immune cells of the choroid. We have used the anti-TRP2 antibody to investigate MB/melanocytes in immunostained choroidal wholemounts from E15.5 to P8 albino Tyr^−/−^ mice, which gave us the opportunity to examine the overall distribution of MB in the entire posterior segment uveal tract. We demonstrate that TRP2^+^ MB are present in the mesenchyme that forms the choroid from the earliest time points examined (E15.5). We describe how MB become more numerous in the first week of postnatal life (broadly equivalent with the third trimester in humans) as the vasculature matures, and subsequently form a dense multilayered network beneath the developing choriocapillaris and surrounding the deeper vasculature. In the developing choroid, the MB did not seem to have a particular association with nerves or cells of the myeloid lineage. The data support our hypothesis that melanocytes arise from MB that differentiate within the developing neural crest-derived mesenchyme that envelops the optic cup, and that they do not invade the choroid as late in development as previously believed.

## Methods

### Mice

Timed matings between naïve B6(Cg)-Tyr^c-2J^/J, C57BL/6J mice that carry a mutation in the tyrosinase gene, rendering them albino, were used to investigate the developing choroid in E15.5 (*n* = 2), E18.5 (*n* = 4), P0 (*n* = 7), P2 (*n* = 4), P4 (*n* = 4), P6 (*n* = 4), and P8 (*n* = 4) eyes. Adult dams (*n* = 4) sacrificed at time of collection of prenatal tissues were used as controls. All animals were housed in conventional facilities and maintained on a 12:12 hour light/dark cycle with access to food and water ad libitum. All procedures were approved by the Monash Animal Research Platform Animal Ethics Committee (MARP/2014/074) and performed in accordance with the ARVO Statement for the Use of Animals in Ophthalmic and Vision Research.

### Tissue Collection and Processing

Adult B6(Cg)-Tyr^c-2J^/J, C57BL/6J mice were sacrificed via an intraperitoneal injection of sodium pentobarbital and enucleated eyes were immersion fixed in 4% paraformaldehyde. After dissection of pregnant females, the heads of E15.5 and E18.5 embryos were removed and immersion fixed in 4% paraformaldehyde at 4°C overnight. Postnatal pups were similarly processed. Eyes were dissected from the heads as a complete cup as previously described^13^ to prepare either whole eye cups (for smaller samples) or the lens and retina were removed from eye cups and were either processed intact or in larger eyes radial incisions were made to flatten the choroid-sclera and anterior segment with iris before whole-mount immunostaining. In the case of E15.5, the sample size was originally *n* = 4; however, owing to the technical difficulty of dissecting the choroid/sclera from such small eyes and processing such tiny tissue pieces in immunostaining protocols as well as mounting for confocal microscopy, ultimately, we had only *n* = 2 for quantitative analysis.

Eyelid skin was also collected as control tissue.

### Immunofluorescence Staining and Confocal Microscopy

Tissues were initially washed in PBS, permeabilized in 20 mM EDTA at 37°C for 1 hour, and blocked in 3.0% (w/v) bovine serum albumin (Sigma, St Louis, MO) and 0.3% (v/v) Triton X-100 (ProSciTec, Kirwan, QLD) in PBS with 5% donkey serum for 1 hour at room temperature. Samples were then incubated with primary antibodies; goat anti-TRP2; rabbit anti-Iba-1; isolectin B4-biotin (Ib4), overnight at 4°C (see Table for detailed antibody information). Tissues were washed in PBS, and subsequently incubated with fluorophore-labelled secondary antibodies (donkey anti-goat 488; donkey anti-rabbit 594) and Hoechst 33342 (1:1000) for 2 hours at room temperature. Tissues were again washed and then mounted onto microscope slides and cover-slipped using ProLong Diamond Antifade Mountant (Molecular Probes, Eugene, OR; P36961). To stain with Ib4, samples were incubated overnight at 4°C and subsequent staining with streptavidin-Cy3 before staining with anti-TRP2 as described above. Eyelid skin (Supplementary Fig. 1), adult choroid and iris controls (from B6(Cg)-Tyr^c-2J^/J, C57BL/6J) were processed in parallel with fetal/embryonic at the time points previously detailed.

**Table.** Primary and Secondary Antibody Descriptions, Including Targets and Suppliers

Name (Titer)	Target	Species Raised	Supplier	References*
Primary antibodies and stains				
Anti-TRP2 (1:200)	Melanosome membranes in MB (stages I–II) and melanocytes (stages III–IV)	Goat	Santa Cruz Biotechnology	^14^^,^^15^^,^^16^^,^^17^^,^^18^
Anti-Iba-1 (1:300)	macrophage/microglia	Rabbit	Wako, 019-19747	^4^^,^^19^^,^^20^^,^^21^^,^^22^
Isolectin B4-Biotin (1:100)	Lectin - vascular endothelium and myeloid cells	N/A	Vector Laboratories, B-1205	^4^^,^^21^^,^^23^
Secondary antibodies and stains				
Alexa Fluorophore 488 (1:200)	Goat	Donkey	Molecular Probes, A11055	
Alexa Fluorophore 594 (1:400)	Rabbit	Donkey	Molecular Probes, A21207	
Streptavidin-Cy3 (1:400)	Isolectin B4-biotin	N/A	Molecular Probes, 434315	
Hoechst 33342 (1:1000)	Eukaryotic nuclei	N/A	Molecular Probes, H1399	

*Abbreviations*: TRP2 = Tyrosinase related protein 2; Iba-1 = Ionized calcium binding adaptor molecule 1; N/A = not applicable. *Evidence of previous experience with and validation of the respective primary antibodies by the authors.

Wholemount samples were imaged from the retinal to the scleral aspect with Olympus Y60-BAIR Fv 1000 (Beatson Institute, Glasgow, UK), SP5 (Leica Microsystems, Wetzlar, Germany; Monash Medical Imaging) and Nikon C1 (Nikon Instruments Inc., Monash Medical imaging) confocal microscopes. Images were captured using 20x (Plan Fluor 0.75 numerical aperture multi-immersion), 40x and 63x (Plan Fluor 1.3 and 1.35 numerical aperture oil, respectively) objectives. Z stacks were captured at 0.5 to 2.0 µm and maximum projection images were created using FIJI^24^ and Imaris (Bitplane, Zürich, Switzerland).

### Quantitative Analysis of MB and Melanocytes in the Developing Choroid

An analysis of cell parameters were performed as previously described.^25^ In short, after optical removal of the RPE, a surface was created using the surface tool within Imaris software, allowing for analysis of cell-surface area (mm^2^) and volume (mm^3^) for MB/melanocytes (TRP2^+^) and myeloid (Iba-1^+^) cell populations in the developing choroid.

To quantify cell density, cells were counted using the spots tool within Imaris software with consistent thresholding for cell size in all images assessed, manually confirmed throughout the frames, and presented as cells/mm^2^, a methodology used in several studies.^25^^–^^28^ In instances where the samples presented with folds resulting in the inability of optical exclusion of the RPE layer, manual counting of the cell populations through each image stack of the captured data was performed (Supplementary Fig. 2).

### Statistical Analysis

Results are presented as mean ± SEM, and statistical significance was determined by 1-way ANOVA with a Tukey post hoc test (Prism 8 GraphPad Software, La Jolla, CA) to correct for multiple comparisons. Differences between groups were considered significant at *P* < 0.05.

## Results

### MB Are Present in the Developing Choroid From E15.5 Onward

**Figure 1.**
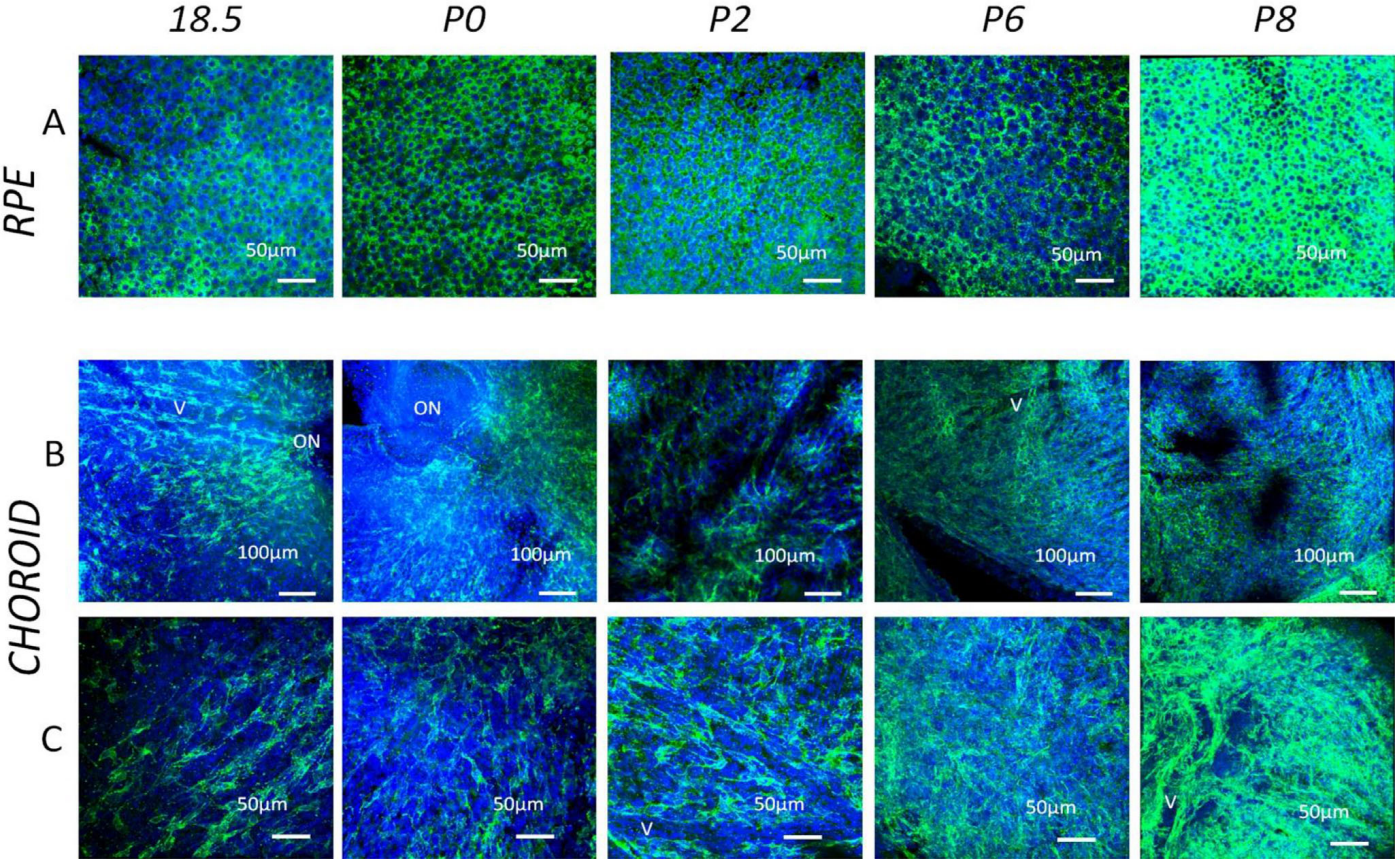
Confocal microscopy images of choroidal wholemounts in the developing mouse eye at various time points (E18.5, P0–P8) stained with anti-TRP2 antibody (*green*) and Hoechst (*blue*). Panel A represents the Z stacks at the level of the RPE alone. This acted as a positive inbuilt control in each immunostaining run as can be seen from the panels the TRP2^+^ granules in the RPE cells. Note the density of TRP2^+^ melanin increases in this monolayer at later stages of development. Panel B and C illustrate low and high-power views of the underlying choroid (more centrally located) respectively (ON = optic nerve head labelled when present in the wholemount). Note the radial incisions used to ensure flattening of the wholemounts are occasionally visible. The density of the TRP2^+^ MB increases with age. In early time points developing choroidal vessels (v) are visible between columns of MB in the intervening developing connective tissue. This indicates that the choroid is thin with only one layer of MB. Note that by P8 there is more than one dense layer of MB/melanocytes, thus obscuring any vascular pattern.

**Figure 2.**
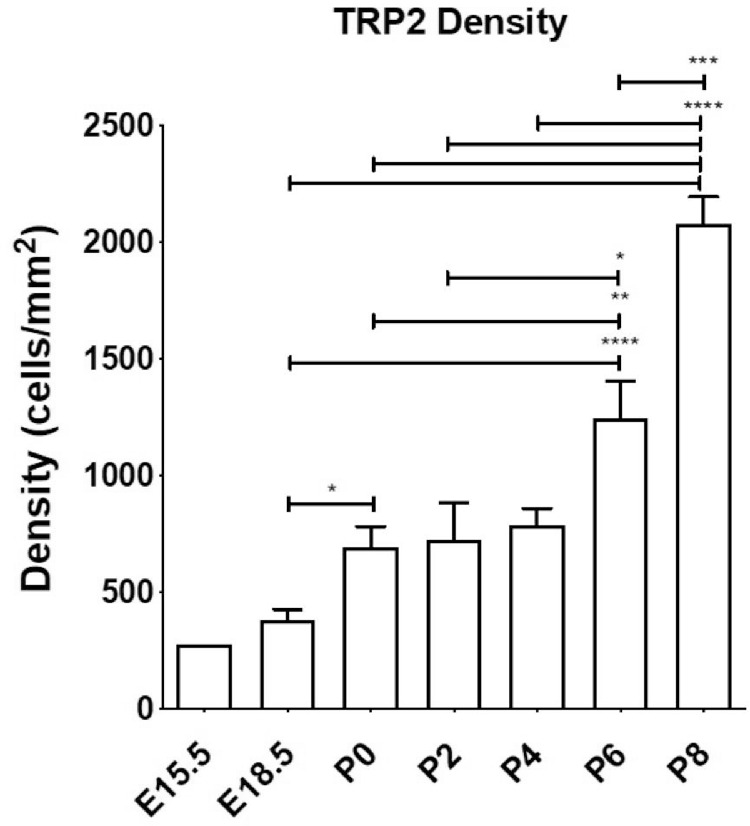
Density of TRP2^+^ cells in the choroid during development as assessed by confocal microscopy. The number of animals per time point were as follows: E15.5 (*n* = 2), E18.5 (*n* = 4), P0 (*n* = 7), P2 (*n* = 4), P4 (*n* = 4), P6 (*n* = 4) and P8 (*n* = 4). ANOVA * *P* < 0.05; ** *P* < 0.01; *** *P* < 0.001.

Staining of the whole-mounted eye cups^13^ from prenatal and postnatal mouse eyes at all time points revealed distinct and consistent staining of the RPE (Fig. 1A), indicating that the antibody TRP2 reacts with melanosomes, thus acting as an internal control. This finding was further supported by positive control tissue (ear skin), which was processed in parallel with the eye cups and also showed staining of TRP2^+^ melanocytes in the epidermis at all stages (Supplementary Fig. 1). Confocal analysis of stained posterior segment wholemounts (minus retina) at E15.5 revealed a few MB in the tissue deep to the RPE (Fig. 4A, Supplementary Video 1), but these cells became more conspicuous and numerous by E18.5, P0, and older (Figs. 1B, 1C; Supplementary Video 2; P2), where they gradually formed a multilayered network in which it was difficult to distinguish individual cells owing to the density of melanocytes (see P6 and P8) (Figs. 1B, 1C). Although at early prenatal time points there was a slightly higher MB density at the posterior portion of the choroid (close to optic nerve), this became less evident from P0 onward (Figs. 1B, 1C). This qualitative impression of increased MB density with age was supported by quantitative analysis in which significant changes in density occurred around P0 (695.2 ± 87.1 cells/mm^2^; *P* = 0.032), and at P6 (1248.0 ± 156.7 cells/mm^2^; *P* < 0.001), compared with E18.5 (Fig. 2).

### The Relationship of MB to the Developing Choroidal Vasculature

**Figure 3.**
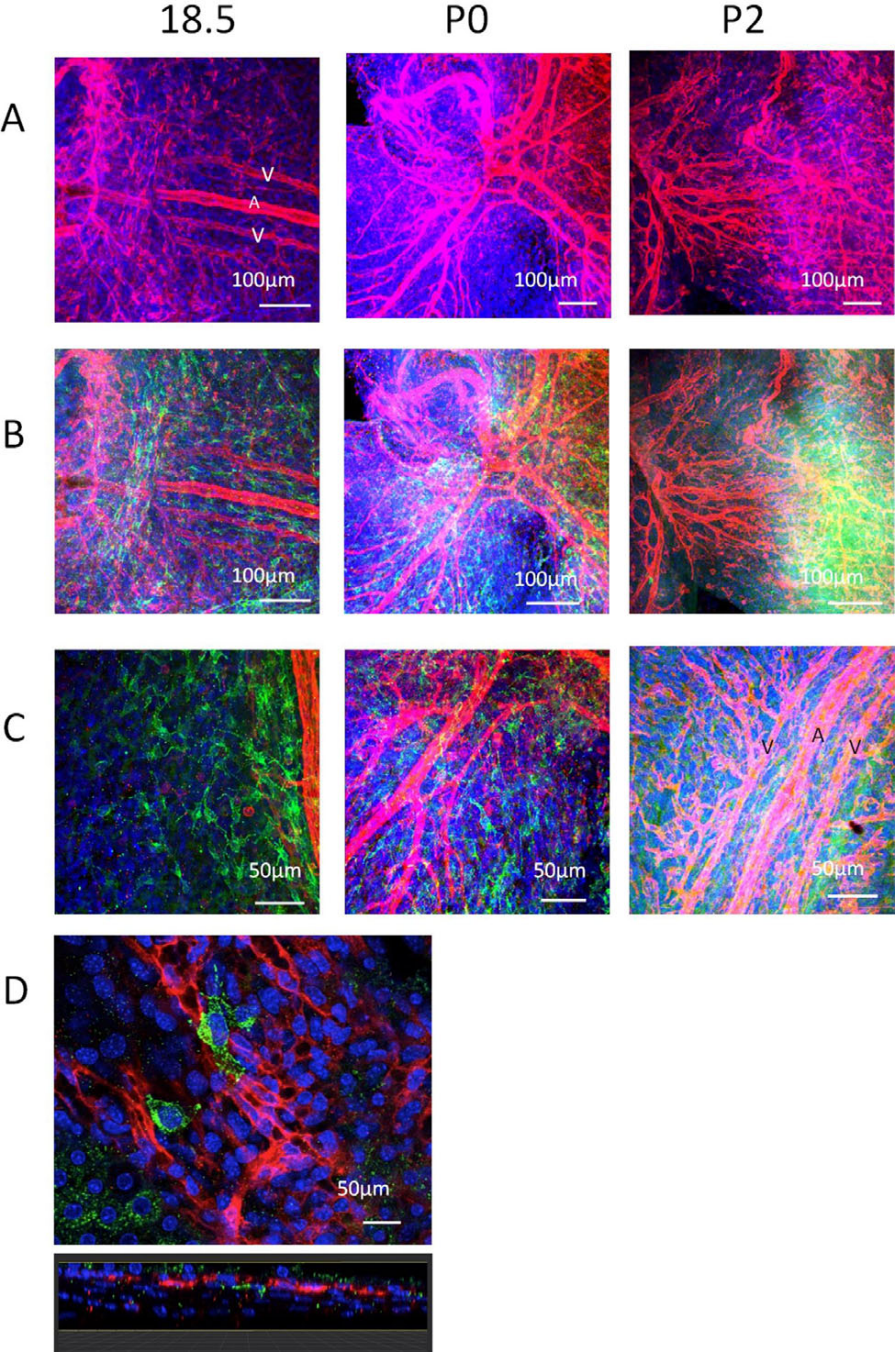
Confocal microscopy images of choroidal wholemounts in the developing mouse eye at early developmental time points (E18.5, P0, and P2) double stained with anti-TRP2 antibody (*green*) and Ib4 (*red*). (**A**) Ib4 staining only to visualize developing choroidal vessels around the optic nerve. (**B**) The same field but with TRP2 staining (*green*) visualized. Note the relationship of MB distribution relative to the choroidal vessels (anteroposterior orientation) and optic nerve (where they assume a circumferential orientation). (**C**) Note the network of MB at E18.5 in the absence of vessels whereas by P2 the choroidal vessels are partially ensheathed by melanocytes. (*D*) High-power view of a few TRP2^+^ MB at E18.5 beneath the Ib4^+^ endothelial cells of the vasculogenic buds of the developing choroidal capillary network. Counterstained with Hoechst (*blue*).

Double staining with Ib4 and TRP2 revealed the extensive nature of the developing choroidal vasculature, even as early as E15.5 where vessels formed in the mesenchyme around the developing optic nerve head. Vasculogenic cords of Ib4^+^ vessels were evident at E15.5 (data not shown), E18.5, and P0 (Fig. 3A), close to the posterior portion of the developing eye. These seemed to extend anteriorly. The posterior ciliary arteries were particularly conspicuous as were paired veins, which seemed to often lie parallel to the arteries (venae commitantes) (Figs. 3A–C; Supplementary Fig. 3), although no particular steps were made to discriminate between arteries and veins in this study. By P2 onward, the larger choroidal vessels seemed mature, although the choriocapillaris or the capillary bed beneath the RPE was only commencing development in the region close to the posterior pole of the eye (Fig. 3D). The MB identified at E15.5 and E18.5 were not closely associated with the larger vessels (Figs. 3C and 3D), even seeming to be quite separated at E18.5 when vessels had still not formed in many areas (Fig. 3C). However, the MB at P2 onward were closely associated with differentiating Ib4^+^ capillaries of the choriocapillaris, tending to be located primarily on the scleral aspect of this layer (see z-profile in Fig. 3D and Supplementary Video 2).

### Myeloid-Derived Cells Are Early Occupants of the Developing Choroid Alongside MB

**Figure 4.**
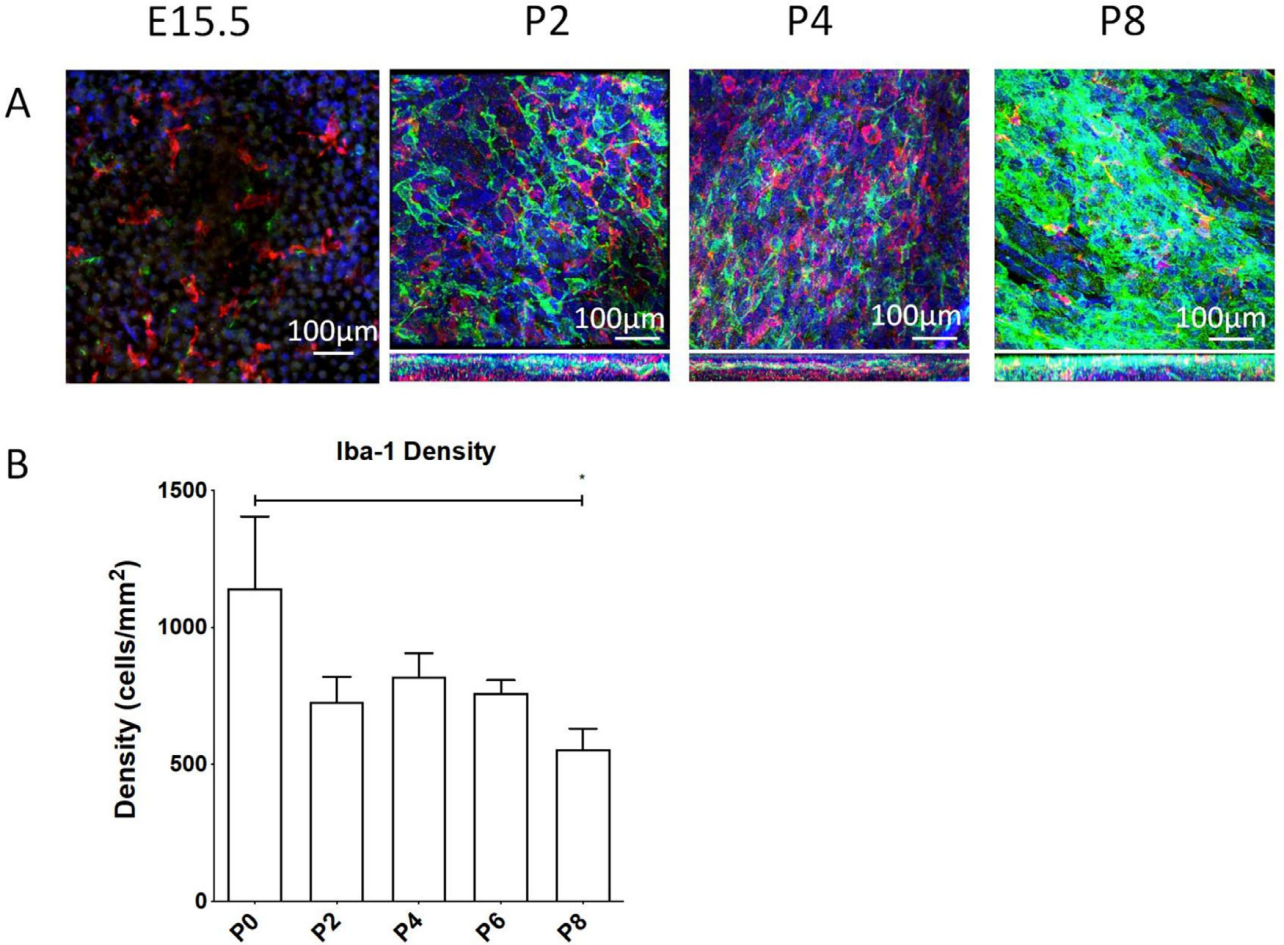
Development of myeloid derived cells in the mouse choroid. (**A**) Confocal microscopy images of choroidal wholemounts in the developing mouse eye at various developmental time points (E15.5, P2, P4, and P8) double stained with anti-TRP2 antibody (*green*) and Iba1 (*red*). Z-profiles are shown for P2, P4, and P8. Note that Iba1^+^ myeloid cells are widespread in the developing choroid even from the earliest time points and indeed quantitative analysis of the density of Iba1^+^ cells. (**B**) The density of these cells in the postnatal period (P0–P8). Insufficient appropriately stained samples were available at prenatal periods to allow quantitation. * ANOVA *P* < 0.029.

At early time points Iba-1^+^ myeloid cells could be identified in the developing choroid, being observed in greater densities than MB at E15.5 (Fig. 4A; Supplementary video 1), E18.5, and P0 (data not shown). However, in later postnatal eyes MB quickly came to outnumber the myeloid cells, which were more scattered as individual cells (Fig. 4A; Supplementary video 2). This density change was confirmed in quantitative analysis (Fig. 4B), which was only possible in P0 eyes onward owing to technical issues of dissecting and isolating the choroid from such small eyes. Density values do not determine total numbers, because no account was taken for growth of the total area of the choroid from the early to the later time points. A qualitative association between myeloid cells and MB was not observed.

## Discussion

**Figure 5.**
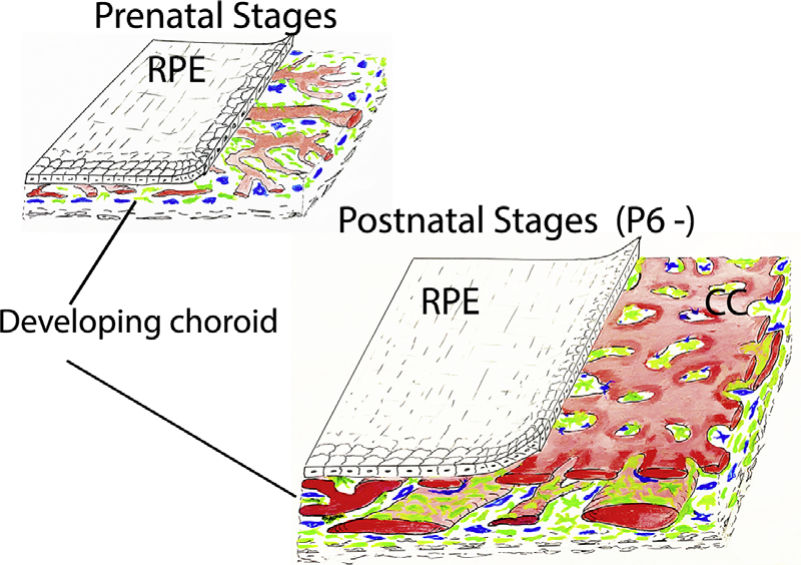
Diagrammatic summary of the changes between early prenatal developing mouse choroidal wholemounts and later postnatal eyes. *Green* = MB/melanocytes (TRP2); *blue* = myeloid (Iba1+) cells. Note the increasing complexity of the vasculature and thickness of the choroid and the marked increase in density of MB in the choroid with age. CC = choriocapillaris.

In this study of choroidal MB in the developing mouse eye, we show using wholemounts and confocal microscopy that MB populate the embryonic choroid earlier than previously described (E15.5), before the appearance of the vascular elements, and that they become multi-layered beneath the choriocapillaris from P2 onwards. Further, this study demonstrates for the first time that Iba-1^+^ myeloid cells are also present at this very early stage in the mouse choroid during embryonic development. Our observations are summarized in diagrammatic form in Figure 5.

Despite the well-defined pathway of cutaneous MB migration,^29^ there is limited information about the development of choroidal MB.^8^^,^^30^^,^^31^ Inexplicably, the exact origin, migration, and maturation timeline of choroidal MB is under-researched, despite the importance of choroidal MB and melanocytes in ocular health and disease. It has been widely suggested that mature choroidal melanocytes do not appear in humans until approximately 28 weeks of development.^6^^–^^8^ This coincides with the time when the human choroid becomes pigmented, that is, when melanocytes start synthesizing melanin.^8^ We hypothesized that MB migrated into the presumptive choroid at an earlier developmental stage, where they mature into later into melanin-bearing melanocytes in situ, similar to that which occurs in the skin.

Although our current study has been conducted in mice, the mouse model has been used as an analogue for human development because the melanogenic pathways are highly conserved across mammalian species.^32^ We found TRP2^+^ MB in the presumptive choroid of mice at our earliest time point of E15.5, which is comparable with approximately 8 to 9 weeks of development in humans, and in increased numbers by E18.5, equivalent to weeks 15 and 16.^33^ The presence of TRP2^+^ cells within the presumptive choroid at E15.5 strongly suggests that melanosome-bearing MB are present within the choroidal mesenchyme at very early stages of eye development. These findings contradict previous reports stating that most melanosomes in choroidal melanocytes are produced postnatally.^34^ For example, Lopes et al.^34^ observed low numbers of melanosome-bearing MB in the P1 mouse choroid, and did not see them at all at E14. However, these authors used cryo-immuno electron microscopy to study tyrosinase and pmel17 expression, not *TRP2*, to determine the presence of choroid melanosomes. Tyrosinase is not expressed in the earliest stage of melanosome formation (stage I), although both TRP2 and pmel17 are expressed or present.^35^

Various types of albinism are known to be linked to development defects in humans, including abnormal decussation, retinal ganglion cell maturation, and fovea–macula formation.^36^ In this study, we made use of *B6(Cg)-Tyrc-2J/J *albino mice, a tyrosinase knockout mouse on a B6 background. The human equivalent disorder, oculocutaneous albinism type 1A, is caused by a mutation in *TYR* gene located on chromosome 11q14.2 encoding tyrosinase.^37^ This mutation leads to a completely inactive or incomplete tyrosinase enzyme polypeptide, and the melanocytes contain no melanin, as without this enzyme the melanin biosynthetic pathway is blocked. Nystagmus may be present at birth or develop later in life, and there is a decrease in visual acuity caused by foveal hypoplasia. The *B6(Cg)-Tyrc-2J/J *albino mice, like the human equivalent, do not lack melanocytes, as is evident by this and many other studies^36^^,^^37^; instead, they lack the enzymes required in the latter stages of melanogenesis. Because the mouse has no macula, fovea, or area centralis and possesses monocular vision with few uncrossed fibers,^38^ the retinal abnormalities and abnormal optic nerve decussation defects, described in severe forms of human albinism, are unlikely to be present in the B6(Cg)-Tyrc-2J/J albino mice and to affect the development of MB migration in the choroid, as described in the present study. There are no reports on the Jackson website or mouse phenome database (https://www.jax.org/strain/000058) to suggest any links to diseases/defects similar to the human equivalent disorder.

Recent studies show that cranial melanocytes can arise from at least two different cellular sources: initially from nerve-associated SCP and later directly from the NC.^39^ Although SCP are in themselves derived from NCC, they represent a second source for MB generation. A large proportion of the earliest cranial MB in the E9.5 mouse embryo is in fact SCP-derived and associated with cranial nerves IX to X. In contrast, the later appearing NCC-derived MB are found in E10.5 at the midbrain–hindbrain region. Although the existence of SCP-derived melanocyte populations has been confirmed in both truncal and cranial melanocytes, it remains unresolved whether choroidal MB are derived solely from NCC or if they also arise from SCP. The study by Adameyko et al.^39^ did not clearly demonstrate any SCP-associated MB close to cranial nerve II, the potential origin or migratory pathway of choroidal MB. However, it was recently hypothesized that choroidal MB at the very least use the developing optic nerve (cranial nerve II) and ciliary nerves (predominantly ophthalmic division of cranial nerve V) as supportive ballasts to enter and populate the eye.^40^

Although we demonstrated an increased density of migrating MB in the peripapillary region of the embryonic choroid, we did not observe a perineural affiliation of these; rather, they tended to be perivascular, particularly from E18.5. Unfortunately, our attempts to co-stain MB and nerves (with the lectin wheat germ agglutinin) to highlight the developing nerves and any association between these structures, were largely inconclusive. Hence, our results cannot completely preclude choroidal MB being associated with nerves of the developing choroid. Further investigations with more specific neural markers will be necessary to elucidate whether there is a switch from a nerve to vasculature association, or if there is an association between the three codeveloping networks.

Three “waves” of NCC migration have been documented in human embryology, although it has been suggested there are only 2 waves in mice.^41^ Previous reports hypothesize that cutaneous MB can populate the embryo throughout these waves, appearing at both early (E10.5) and late (E16.5) stages of NCC migration.^39^^,^^42^ When explored in mice carrying pigmentary defects, this late migratory MB wave almost repopulated the entire trunk.^42^ One plausible mechanism for MB migration to the choroid might be a self-generated gradient,^43^ which would predict a wave of cells arriving at the same time. However, we did not observe any such wave-like migration of MB in the mouse eye. It seems, instead, that they enter the eye following migration from the initial MB populations derived from the NC; the attractants for their migration remain unknown, but may include developing cells within the choroid or retina, including the choroidal fibroblasts or RPE.

Our demonstration of MB in the presumptive mouse choroid before complex vasculature had formed is novel. Further, we showed that the MB are closely associated with the sclerad aspect of the choriocapillaris as its development progresses. The signals that prevent MB (or any cells) accessing the space between the basement membrane of the choriocapillaris and the outer collagenous zone of Bruch's membrane, which is essential for the physiological role of this dense capillary bed, are unknown. The development of the human choroidal vasculature has been subject to detailed morphological studies^44^^,^^45^ and recently reviewed by Saint-Geniez and D'Amore.^46^

It has been proposed that uveal melanocytes are required for healthy vasculature in the adult choroid, because the vasculature is ectopic in *Mitf^mi-bw^/Mitf^mi-bw^*(melanocyte-deficient) mice.^47^ Further, in these transgenic mice, where the RPE does not properly form, both the vasculature of the choroid and the melanocytes do not migrate throughout the choroid, with a few populations remaining adjacent to the optic nerve.^48^ It is well-known that the RPE secretes VEGF,^49^ which may be an important regulator of choroidal blood vessel formation and, therefore, providing a route/pathway by which the MB can migrate. Although we have shown MB in the presumptive choroid, a limitation of our study was the inability to detect expression of MITF and SOX10 using immunostaining protocols (results not shown). Because Dopachrome Tautomerase/TRP2 is expressed in the membrane of melanosomes (stages I–IV),^11^ and does not highlight primitive/presumptive MB-lacking melanosomes, we may have missed specific populations of SCP–melanogenic precursors that had not committed to a melanogenic fate via the production of melanosomes.

There are striking similarities between normal organogenesis and tumorigenesis. The close association of the choroidal MB with the developing choroidal vasculature may have relevance in understanding the behavior of malignant choroidal melanocytes, that is, UM and their ability to produce complex intratumoral vasculature and to disseminate haematogenously.^50^ Highly metastatic UM demonstrate an epithelioid cell morphology with complex connective tissue loops containing vascular structures, scattered immature vascular lakes, as well as genetic alterations including loss of one copy of chromosome 3, amplifications of chromosome 8 and mutations in *BAP1.*^51^^–^^53^ Such aggressive UM have stem cell-like properties, enabling their migration and dissemination.^54^^,^^55^ Therefore, by understanding the mechanisms involved in choroidal melanocyte biology during development novel insights into the biological pathways regulating UM may be delineated. To this end, genetically engineered mouse models have been developed to study mouse MB migration.^56^ The application of new molecular techniques (e.g., single cell sequencing and RNAseq) as well as the study of MB cell replication and ultimate differentiation into melanocytes may enable the determination of specific cell populations and processes, as well as transcription factors that are involved in cell proliferation, mobility, and dissemination in cutaneous and noncutaneous MB.^57^

A further major finding in our study was the appearance of Iba-1^+^ myeloid cells—that is, cells of the macrophages/monocyte/dendritic cell lineage—in the presumptive choroid at early stages (E15.5, E18.5, and P0). We show that these cells are present in high densities at prenatal stages with their numbers approaching adult levels in postnatal eyes (see reviews^4^^,^^58^). Most previous research on choroidal myeloid cells has concentrated on their phenotype, function, turnover, and changes with age in the adult eye.^59^^–^^61^ Iba-1^+^ myeloid cells turnover more frequently than retinal microglia, display functional heterogeneity, and have a close association with the choriocapillaris.^62^ Although the function of the choroidal Iba-1^+^ myeloid cells is still an area of active research, especially with regard to aging changes and the pathogenesis of AMD, our findings demonstrate their early appearance in the uveal tract coinciding with the formation of the choroidal vascular network, similarly to the choroidal MB. The relationship between MB and Iba-1^+^ myeloid cells is unclear; further work is required to determine if they interact during development or even in adulthood, because these cells have the potential to be key components of maintaining choroidal integrity.

In summary, we have demonstrated that MB are present from the earliest stages of choroidal development, with a clear association with the vasculature emerging as the eye further matures. MB migrate into the choroid earlier than previously appreciated; the precise factors controlling the differentiation of NCC into choroidal MB and SCP in the periocular mesenchyme are unknown. The previously held view that in the developing mammalian eye, melanocytes migrated into a near mature choroid is no longer tenable based on our data.

## Supplementary Material

Supplement 1

Supplement 2

Supplement 3

Supplement 4

Supplement 5

## Supplementary Materials

**Supplementary Video S1: E15.5.** The video commences with a compressed stack of the immunostained RPE and choroidal wholemount viewed from the apical aspect with the Trp2+ (green) RPE monolayer en face. The 3D reconstructed RPE/choroidal wholemount is rotated to show the choroidal aspect more clearly including the occasional or rare Trp2^+^ (green) melanoblasts along with the Iba1^+^ (red) myeloid cells (00:11). The individual Z-slices of the reconstructed choroid wholemount are then shown to highlight the apical Trp2^+^ RPE monolayer with its distinctive evenly spaced nuclei (blue) (00:22–00:24) as well as the underlying thin choroid containing scattered Iba1^+^ (red) myeloid cells and small less common Trp2^+^ melanoblasts (green) (00:25–00:28). The stack is then rotated and a lateral orthogonal ‘fly-through’ is shown with all channels visible (00:40–00:57), and repeated to highlight Trp2^+^ (green, 00:58–01:16) and Iba1^+^ (red, 01:17–01:34) myeloid cells. These perspectives confirm the location of rare scattered melanoblasts in the thin choroid beneath the RPE.

**Supplementary Video S2: P2.** The video commences with a compressed stack of the immunostained RPE and choroidal wholemount viewed from the apical aspect of the Trp2^+^ (green) RPE monolayer en face and rotated to show distribution of cells throughout the choroidal aspect of the wholemount. Due to the large numbers of Trp2^+^ melanoblasts (green) at this time point, the distinctive evenly spaced nuclei (blue) morphology of the RPE is only made clear once the reconstructed choroid is examined in Z-series steps (00:23–0:25). Moving past the RPE through the reconstructed choroidal wholemount the more substantive choroid (compared to the E15.5 time point in Video 1) is illustrated showing scattered Iba1^+^ (red) myeloid cells and large numbers of complex shaped Trp2^+^ melanoblasts (green) many of which are orientated parallel to and surrounding vessels (00:25–00:29). Due to the high density of melanoblasts (green) which dominate the field of view the numbers of scattered Iba1^+^ (red) myeloid cells appear less significant. As in Supplemental Video 1, the stack is then rotated and a lateral orthogonal ‘fly through’ is shown with all channels visible (green, red, blue) (00:42–00:56), and repeated to highlight Trp2^+^ (green, 00:58–01:14) and Iba1^+^ (red, 01:15–01:34) myeloid cells. These perspectives confirm the location of large numbers of melanoblasts in the P2 choroid beneath the RPE. Both sets of scans were captured using the x40 objective. Scale bars are shown.
