# Supplementary material for: Melanoblasts Populate the Mouse Choroid Earlier in Development Than Previously Described
Source: Invest Ophthalmol Vis Sci. 2020 Aug 14;61(10):33. doi: 10.1167/iovs.61.10.33 (PMC7441366; doi:10.1167/iovs.61.10.33)
Supplement: Supplement 1 [file iovs-61-10-33_s001.pdf]

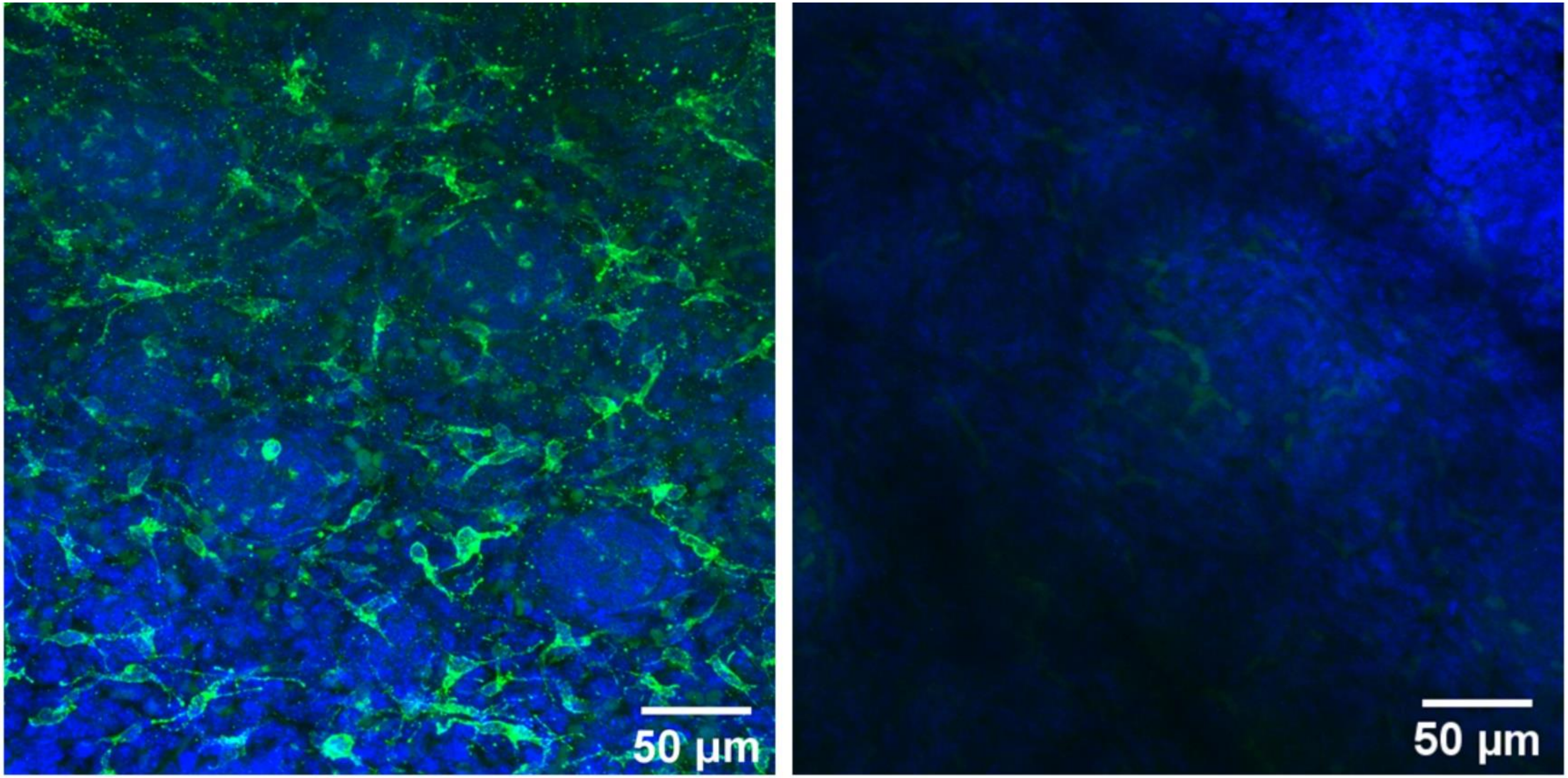

+ -

Figure 1 – Supplementary: Control skin eyelid stained with TRP2 (positive) or with secondary antibodies only (negative).
