# Supplementary figures and images for: Melanoblasts Populate the Mouse Choroid Earlier in Development Than Previously Described
Source: Invest Ophthalmol Vis Sci. 2020 Aug 14;61(10):33. doi: 10.1167/iovs.61.10.33 (PMC7441366; doi:10.1167/iovs.61.10.33)

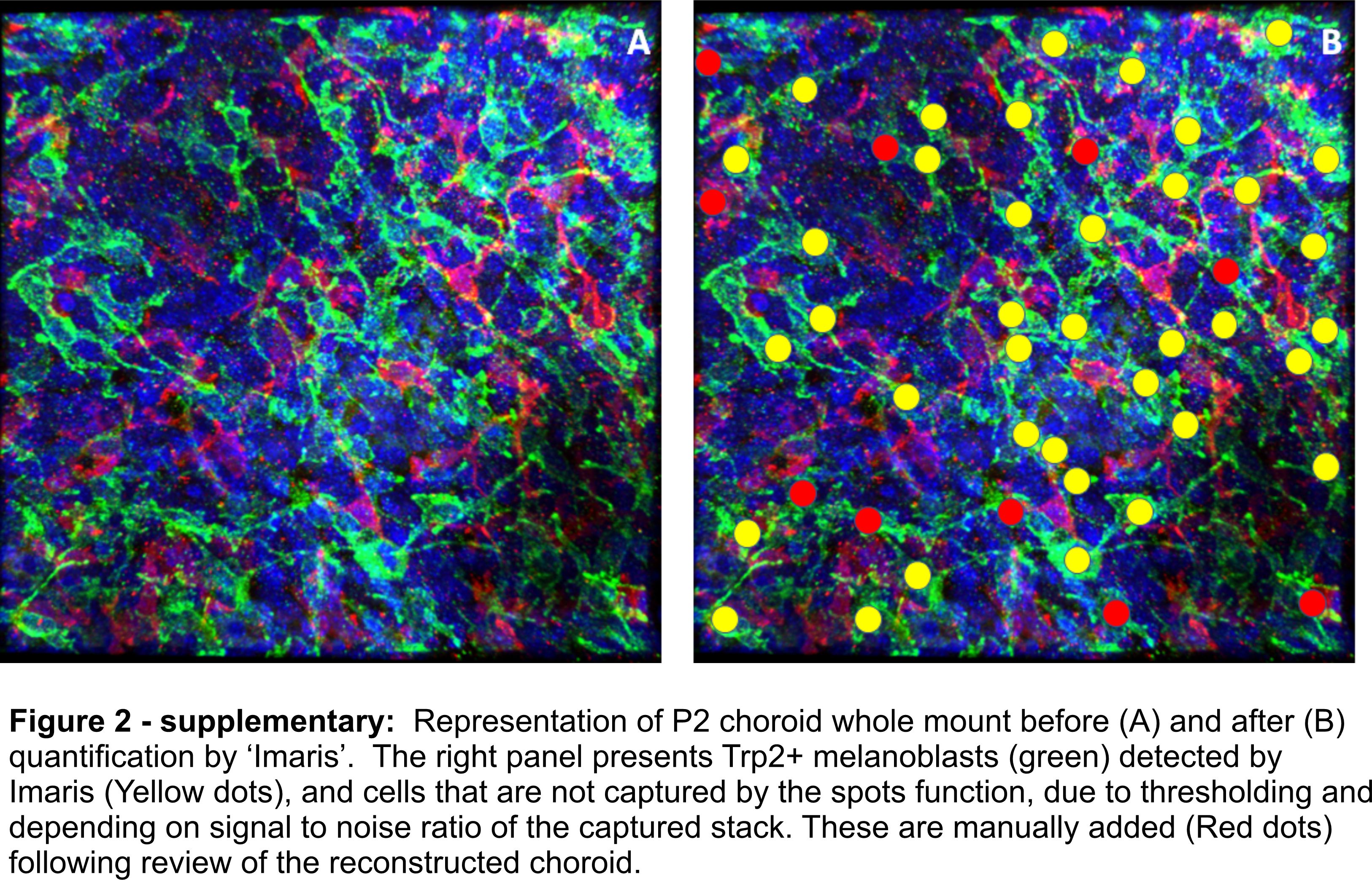

Supplement: Supplement 2 [file iovs-61-10-33_s002.jpg]

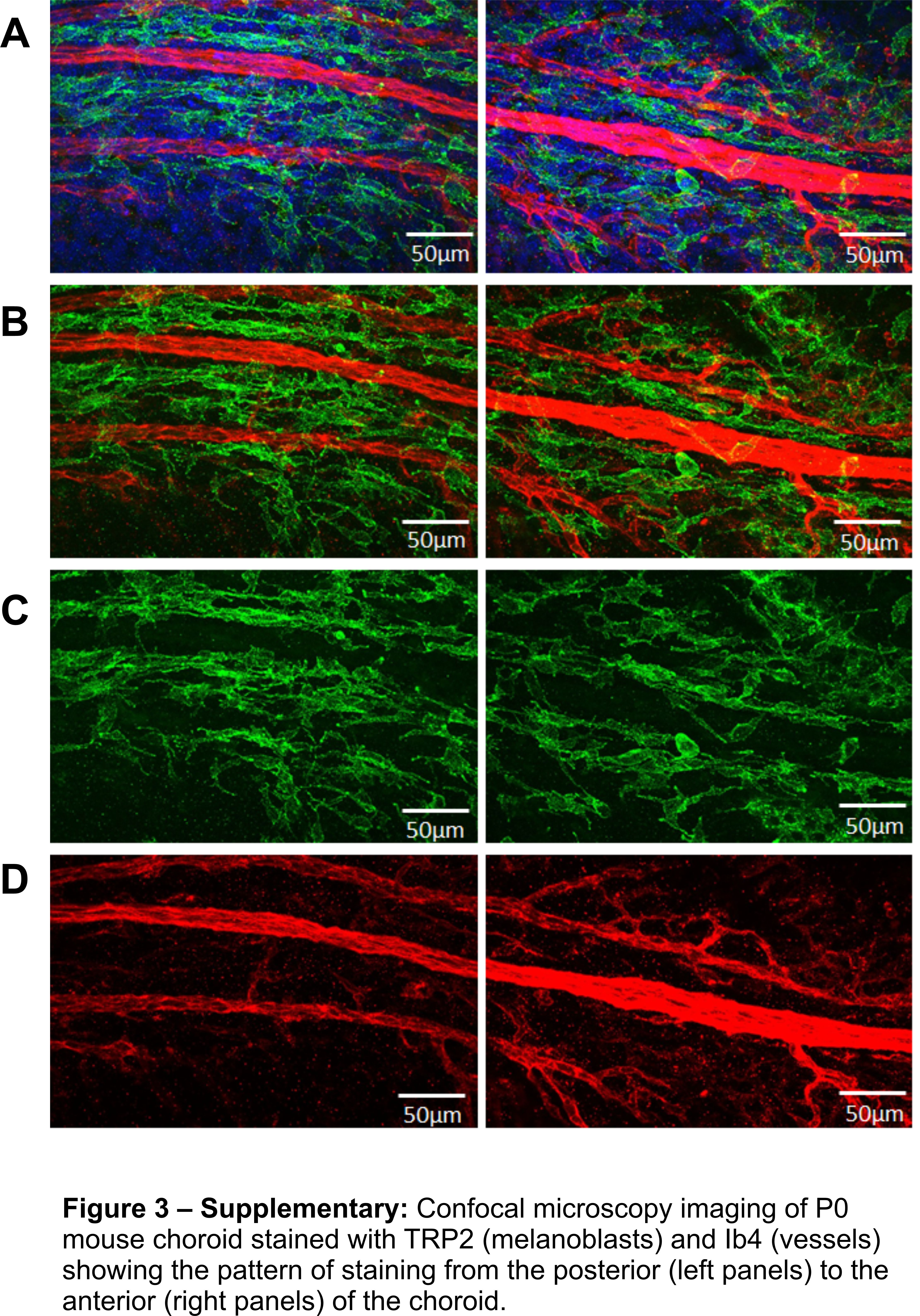

Supplement: Supplement 3 [file iovs-61-10-33_s003.jpg]
